# 

**Published:** 1920-01-31

**Authors:** 


### THE HOSPITAL

### The Modern Newspaper of Administrative Medicine and Institutional Life.

Telegraphic Address : OSPEDALE, RAND, LONDON. Telephone Number: 2734 GERRARD

No. 1756, Vol. LXVII. | Saturday, January 31st, 1920. | PRICE TWOPENCE

